# Supplementary material for: Partitioning of Persistent Organic Pollutants between Adipose Tissue and Serum in Human Studies
Source: Toxics. 2022 Dec 31;11(1):41. doi: 10.3390/toxics11010041 (PMC9866963; doi:10.3390/toxics11010041)
Supplement: Supplementary file 1 [file toxics-11-00041-s001.zip › toxics-2104705-supplementary.pdf]

Supplemental Materials

Partitioning of persistent organic pollutants between adipose tissue and serum in human studies

Table S1. Search strings

**Scheme 05.**
“(TITLE-ABS-KEY(“Persistent Organic Pollutants”) OR TITLE-ABS-KEY(“Polybrominated diphenyl ethers”) OR TITLE-ABS-KEY(“polychlorinated biphenyls”) OR TITLE-ABS-KEY(“Halogenated Diphenyl Ethers”) OR TITLE-ABS-KEY("Hexachlorobenzene") OR TITLE-ABS-KEY("Hexachlorocyclohexane") OR TITLE-ABS-KEY("Dioxins and Dioxin-like Compounds") AND TITLE-ABS-KEY(“human”) AND (TITLE-ABS-KEY(“adipose”) OR TITLE-ABS-KEY("Adipose Tissue")) AND ((PUBYEAR > 2011))”
PUBMED (05/08/2022)
(“Persistent Organic Pollutants”[Text Word] OR “Polybrominated diphenyl ethers”[Text Word] OR “polychlorinated biphenyls”[Text Word] OR “Halogenated Diphenyl Ethers”[Text Word] OR Hexachlorobenzene[Text Word] OR Hexachlorocyclohexane[Text Word] OR "Dioxins and Dioxin-like Compounds"[MeSH Terms] AND “human”[Text Word] AND (“adipose”[Text Word] OR “Adipose Tissue”[MeSH Terms]) AND ("2011/01/01"[Date - Publication].
